# Case Report: Analysis of Plasma Extracellular Vesicles in a Triple Negative Spindle-Cell Metaplastic Breast Cancer Patient

**DOI:** 10.3389/fmed.2022.827206

**Published:** 2022-03-09

**Authors:** Ivan Vannini, Milena Urbini, Mattia Melloni, Tania Rossi, Giulia Gallerani, Michela Palleschi, Irene Azzali, Maurizio Puccetti, Giovanni Martinelli, Francesco Fabbri

**Affiliations:** ^1^Biosciences Laboratory, IRCCS Istituto Romagnolo per lo Studio dei Tumori (IRST) “Dino Amadori”, Meldola, Italy; ^2^Laboratory of Biomarkers, Biomolecular Targets and Personalized Medicine in Oncology, Translational Medicine, University of Ferrara, Ferrara, Italy; ^3^Department of Medical Oncology, IRCCS Istituto Romagnolo per lo Studio dei Tumori (IRST) “Dino Amadori”, Meldola, Italy; ^4^Unit of Biostatistics and Clinical Trials, IRCCS Istituto Romagnolo per lo Studio dei Tumori (IRST) “Dino Amadori”, Meldola, Italy; ^5^Azienda Unità Sanitaria Locale Imola, Imola, Italy; ^6^Scientific Directorate, IRCCS Istituto Romagnolo per lo Studio dei Tumori (IRST) “Dino Amadori”, Meldola, Italy

**Keywords:** metaplastic breast cancer, extracellular vesicles, next generation sequencing (NGS), plasma, metastasis

## Abstract

Metaplastic breast cancer (MpBC) is a rare tumor representing 1% of all breast malignancies. The prognosis of this histologic subtype is actually poor and there are no current clear-cut therapeutic guidelines. Hence, despite its uniqueness, its aggressive prognostic profile strongly encourages further studies to identify new markers and therapeutic targets. Herein, we report a case of 32-years-old patient affected with of triple negative spindle-shaped MpBC. The research of molecular targets on the primary tumor did not allow performing an effective therapeutic choice. Extracellular Vesicles (EVs) are under intense study as new potential pathophysiological markers and targets for therapeutic applications, in different tumors for their role in tumor onset, progression and aggressiveness. Here, we examined the involvement of EVs in this case, to look into the MpBC microenvironment willing to identify new potential molecular targets, pathways of aggressiveness, and markers of prognosis and therapeutic efficacy. Firstly, we characterized MpBC patient EV dimensions and surface proteins. Moreover, we analyzed the EV RNA cargo supposed to be delivered to nearby and distant recipient cells. Interestingly, we observed a dysregulation EV-contained miRNAs, which could determine an increased expression of oncogenes in the tumor microenvironment, probably enabling cancer progression. These data suggest that the characterization of miRNA cargo of EVs could be important for the identification of new markers and for the application of future new target therapies.

## Introduction

Metaplastic Breast Cancer (MpBC) is a unique subtype of breast cancer, infrequent tumor (<1%) but severely aggressive. It is a triple negative ductal carcinoma that shows intra-tumoural heterogeneity that has epithelial differentiation into squamous and/or mesenchymal elements, with often co-existing cells that display a spindle, squamous, chondroid or osseous transformation (1). The World Health Organization Classification groups MpBCs in different subtypes depending on the metaplastic features (2–5): squamous cell, fibromatosis-like metaplastic, low grade adenosquamous, spindle cell, metaplastic with mesenchymal differentiation, mixed metaplastic, and myoepithelial carcinomas, all similarly treated ineffectually in the clinical setting. Indeed, MpBCs do not respond to hormone and anti-HER2 therapies, and to conventional chemotherapies, generating the worst prognosis among breast cancer types. A dysregulation of different molecular pathways that are involved in cell proliferation or epithelial-to-mesenchymal transition (EMT), has been observed in MpBC patients (4), but the scarcity of recognizable effective therapeutic targets has halted the improvement of the prognosis. These elements explain the urgent need to research for new markers that allow the identification of the most suitable therapeutic choice. Extracellular vesicles (EVs) are membrane-bound lipid bilayer structures secreted by almost all cells. They are a heterogeneous class and they form mainly from either endosomal multivesicular bodies (exosomes) or the plasma membrane (microvesicles). Exosomes have a size around 50–200 nm, microvesicles range from around 150 to 1000 nm (6–10). EVs express also a number of surface markers, among those tetraspanins CD9, 63, and 81 are probably the most known. However, since there is quite an overlap in terms of markers and dimensions, a more correct classification has to refer to small EVs and large EVs, without more specific definitions. To form a pre-metastatic niche and to induce migration, invasion, and drug resistance in disseminated cancer cells the role of EVs is thought to be essential, since they have been implicated in tumor onset, progression, metastasis, and drug resistance. Indeed, involved in microenvironment cell-to-cell communication, they transfer DNA, RNA and proteins regulating intra-and inter-cellular events (11–15). In addition, EVs are present in different types of fluid bodies such as cerebrospinal fluid, blood plasma, serum, saliva and urine. This allows the identification of potential diagnostic and therapeutic markers in a non-invasive and cost-effective way. For these reasons, EVs have been studied a lot in recent years (10, 16–18) and are an option worthy of being investigated to find new biomarkers of prognosis and prediction of sensitivity or resistance to therapy. In a recent study, we analyzed the circulating tumor cells in a patient with MpBC, discovering chromosomal alterations that may have a role in the metastatic cascade (19). Considering the rarity of the disease and the resistance to available therapies, we decided to deepen the case by studying the EVs. We isolated them from the plasma of the patient with MpBC *via* size exclusion chromatography (SEC) and we compared EVs with those of 3 patients with non-metaplastic metastatic breast cancer who presented a better prognosis. We investigated their size, membrane surface proteins and RNA cargo. We observed that the increased aggressiveness of the MpBC patient could be due to a specific RNA cargo dissimilar from that of better-prognosis metastatic breast cancer patients.

## Case Presentation

**FIGURE 1:**
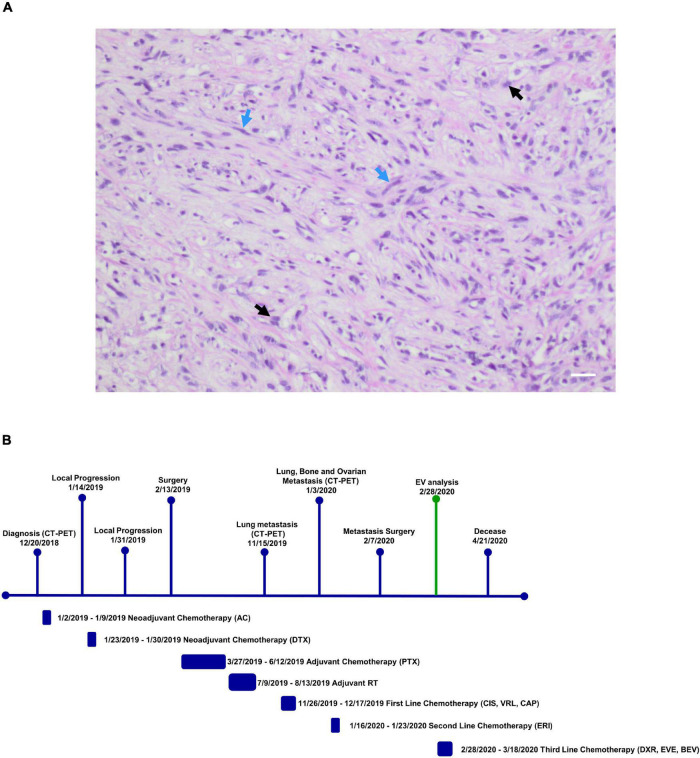
**(A)** Microscopic eosin and hematoxylin image of metaplastic breast carcinoma (MpBC) with spindled (blue arrows) and oval (black arrows) cells. Scale bar = 50 μm. **(B)** Patient timeline. Patient history showing her clinical course above and therapy administered to patient below. CT-PET, computed tomography-positron emission tomography; AC, adryamicin-cyclophosphamide; DTX, docetaxel; PTX, paclitaxel; CIS, VRL, CAP, cisplatin, vinorelbine, capecitabine; ERI, eribulin; DXR, EVE, BEV, doxorubicin, everolimus, bevacizumab; EV analysis, extracellular vesicle analysis.

In this study, we describe a 32-year-old patient with triple-negative spindle-cell MpBC. The disease was diagnosed in December 2018 and a 40 mm lesion was observed in the right breast during the Positron emission tomography/computed tomography examination (CT-PET) without affecting bones and viscera. Neoadjuvant chemotherapy (NAC) with adryamycin (60 mg/m2) and cyclophosphamide (600 mg/m2) intravenously for one cycle started in January 2019. Then the disease progressed, so the NAC was changed to docetaxel for one cycle (23th January 2019). Unfortunately, even with the new NAC the progression continued. The patient was operated by performing a right mastectomy with axillary node removal in February 2019. Through a histological examination, a lesion of maximum 65 mm in diameter, ypT3, ypN0 M0, ER 0%, PgR 0%, HER2-neu negative (score 0) and Ki-67 90% were observed. A microscopic image of the Hematoxylin and Eosin tumor section is shown in Figure 1A. She was treated weekly with paclitaxel (80 mg/m2) for 12 cycles from March to June 2019 and from July to August 2019 she received radiotherapy (total dose 50Gy) to right chest. A 40 × 37 mm lung lesion and other sub centimeter bilateral lung nodules were observed through CT-PET in November 2019. BRCA1/BRCA2 were unaltered on primary tumor, while the expression of Programmed Death-Ligand 1 (PD-L1) was <1%. Two cycles of cisplatin (60 mg/m2; day 1), vinorelbine (20 mg/m2; day 1 and 3) and capecitabine (500 mg thrice a day) were administered to the patient from November to December 2019. Lung, bone and bilateral ovarian progression was observed through CT-PET in January 2020. Moreover, the G1049A PIK3CA mutation and amplification of the MYC locus (copy number: 26 copies) on the primary tumor were shown through the NGS Oncomine Focus Assay (Thermo Fisher Scientific, Waltham, MA, United States). Two cycles of eribulin (1.23 mg/m2) were administrated in January 2020 and bilateral ovariectomy and wedge liver resection were performed in February. Triple negative MpBC metastases and several subcutaneous metastases on the scalp, neck and chest, other than bilateral lung nodules were detected with the histopathology analysis. The physicians prescribed an off-label treatment in the lack of effective therapy: doxorubicin (30 mg/m2) plus bevacizumab (15 mg/kg every 3 weeks) plus everolimus (7.5 mg daily). The treatment began without bevacizumab (due to recent operation) in February 2020. An improvement on pain, a clinical stable disease, and reduction of all subcutaneous nodules were noticed. EV analysis had begun before this last line of treatment. The second cycle with bevacizumab was administered in March 2020. The patient showed no pain, none of the most subcutaneous metastases, and a good quality of life. Cough, fever and low blood pressure complicated her health in April 2020. She was treated with antibiotics and steroids but they were not effective. On 21th April 2020, she deceased of respiratory problems. The Patient’s timeline is represented in Figure 1B.

## Isolation and Analysis of Extracellular Vesicles

**FIGURE 2:**
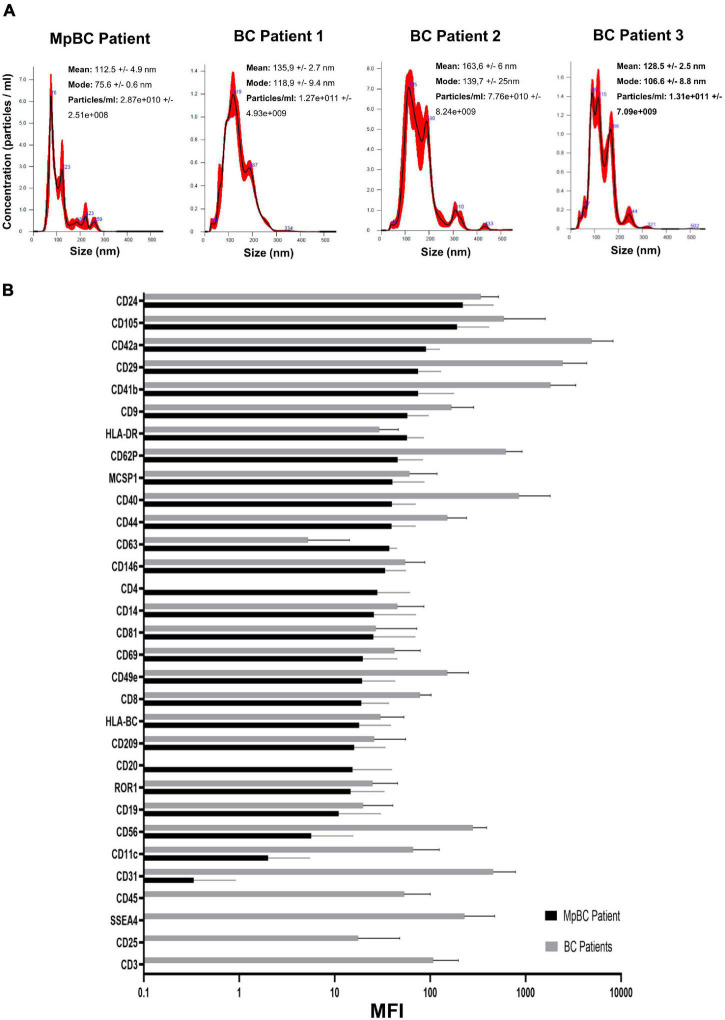
**(A)** NTA profile analysis of SEC fractions with highest concentration of EVs obtained from MpBC patient plasma and 3 metastatic breast cancer patients. **(B)** Protein expression of each plasma EVs marker by flow cytometry. Values refer to Mean Fluorescence Intensitiy (MFI) ± s.d. of the most concentrated fractions. In black, the values obtained from MpBC EVs and in gray the values of 3 Breast Cancer EVs. Values have been normalized to blank control.

**FIGURE 3:**
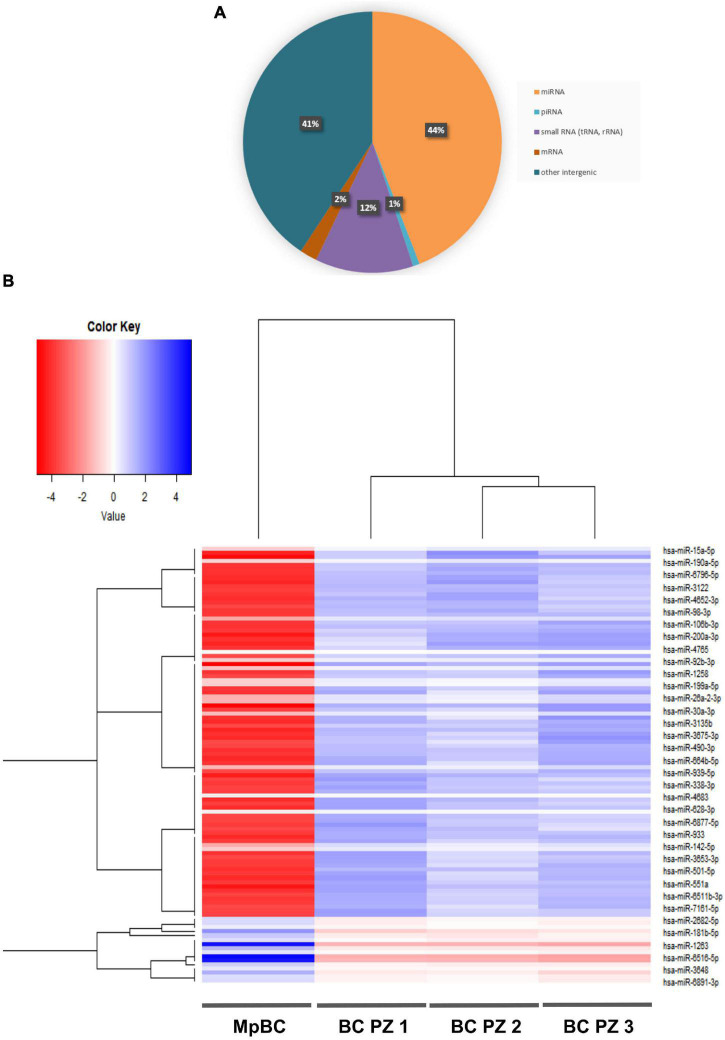
**(A)** RNA species identified through next-generation sequencing in EVs of MpBC case. **(B)** Heat map showing the expression profile of the miRNA contained in EVs. The map shows the differentially expressed miRNAs (*T*-test *q*-value <0.05) in the EVs of MpBC patient plasma compared to miRNA EV cargo of three non-metaplastic metastatic breast cancer patients.

Patient plasma obtained from 5 ml of whole blood prior the administration of the off-label therapy was used for EV isolation. Through Size Exclusion Chromatografy (SEC), 1 ml of plasma was used for the isolation of the EVs. In this technique, a qEV 70 (Izon) column allows the isolation of the EVs through a stationary phase consisting of porous resin particles. By elution, fractions of 500 ul each are obtained. Using the NanoSight NS300 instrument (Malvern Instruments, Malvern, United Kingdom) we characterized vesicle size and concentration. The software used is NTA version 2.3. Figure 2A shows the Nanosight Tracking Analysis (NTA) where we can examine the distribution of vesicle concentration, based on size, mode and mean of the most concentrated fractions in the MpBC patient and in 3 metastatic breast cancer patients studied for comparison. We observed that the most concentrated fraction is the fraction 9 for all tested samples. Subsequently, EVs were characterized for protein surface signature by a protein multiplex bead-based flow cytometry assay as previously reported (20). The median fluorescence intensity (MFI) of each marker is shown in Figure 2B. The typical exosomal markers CD9, CD63, and CD81 were detected. In addition, other surface proteins are expressed such as CD8, HLA-DRDPDQ, CD40, CD62P, CD146, CD42a, CD29 but we observed no significant difference between the markers found in the plasma of the MpBC patient and of the 3 metastatic breast cancer patients. However, four markers were completely absent from the vesicles of the MpBC patient (CD45, CD3, SSEA4, and CD25). Following, we evaluated the miRNA content of EVs of the isolated fractions. Small RNA libraries were produced using Qiaseq miRNA sequencing kit (QIAGEN) and sequenced on Nextseq550 instrument (Illumina). An average of 24 millions of reads per sample were produced. GeneGlobe (QIAGEN) and R Studio RStudio^1^ were used for analysis of small RNA. miRNA and piRNA identified by more than 10 different molecules (UMIs) were considered as expressed. Small RNA abundance was normalized as counts per million (cpm), and the R packages prcomp, stats and gplots were adopted to perform analysis and hierarchical clustering. Approximately 45% of aligned reads cover miRNA and piRNA regions, leading to the identification of 948 miRNA and of 19 piRNA. Other RNA species were detected, including tRNA and rRNA (12% of reads) (Figure 3A). To try to elucidate the origin of the aggressiveness of MpBC, we compared the patient’s EV RNA content with that of 3 patients with metastatic Breast Cancer. Although based on a comparison between very few patients, the reported differences between them are quite striking. miRNA expression profile of the MpBC case differs quite outstandingly from those of other patients. In particular, we identified 106 differential small RNAs (101 miRNA and 5 piRNA) (Figure 3B and Supplementary Material).

## Discussion

The herein presented case regarding a 32 years old patient portrays a representative MpBC resistant to chemotherapy, prone to metastasis, and with a very poor prognosis. However, although clearly deserving better consideration, the rarity of this disease has slowed the research to find effective management strategies. Indeed, a better understanding of the molecular mechanisms that determine MpBC pathogenesis and progression mechanisms is essential to identify new therapeutic targets. To shed some light on this disease, we have characterized the plasma EVs of this patient to discover new biomarkers that are involved in the tumor aggressiveness. Firstly, we compared the size and surface protein expressions of the EVs of MpBC patients with the EVs of 3 non-metaplastic metastatic breast cancer patients finding no significant difference, probably due to the low size of Breast Cancer samples. Thus, we analyzed the EV RNA content, noting that miRNAs are the most expressed RNAs in the all 4 patients. Remarkably, they were differentially expressed between the MpBC patient and the 3 metastatic breast cancer patients: our results showed 106 differentially dysregulated miRNAs in MpBC case. Searching the literature for the role in breast carcinogenesis of the more downregulated miRNAs in the MpBC patient comparison to those observed than in metastatic breast cancer patients, we found some interesting hints. Mainly, we found that downregulated miRNAs in MpBC have a potential tumor suppressor role. *miR-15a-5p* has a tumor suppressor role in BC: long non-coding RNA small nucleolar RNA host gene 12 (SNHG12) enhances cell proliferation, migration and invasion but reduces apoptosis in BC by upregulating the expression of Sal-like 4 (SALL4) and by sponging *miR-15a-5p* (21). Overexpression of *miR-190* in BC inhibited epithelial-mesenchymal transition and angiogenesis by inactivating AKT-ERK signaling pathway through targeting on stanniocalicin 2 gene (*STC2*) (22). *miR-106b* is downregulated too in BC and induces breast cancer cell invasion. Through gain- and loss-of-function studies it has been demonstrated that matrix metalloproteinase 2 (*MMP2*) promotes the migration and invasion of BC cells and *miR-106b* directly regulates *MMP2* expression demonstrating its tumor suppressor role (23). *miR-92b* suppresses viability and invasion in breast cancer through targeting of Enhancer of zeste homolog 2 (*EZH2*) involved in the silencing of tumor suppressors genes (24). *miR-1258* exerts its antitumor action by targeting E2F transcription factor 1 (*E2F1*) (25) while *miR-26a* targets *MCL-1*, an anti-apoptotic member of the Bcl-2 family, determining an inhibition of cell proliferation and migration of breast cancer cells (26). *miR-30a* regulates RTK-like orphan receptor 1 (*ROR1*), a glycosylated type I membrane protein that interacts with the non-canonical Wnt signaling pathway. The targeting on *ROR1* causes a inhibition of epithelial-mesenchymal transition and metastasis in triple-negative breast cancer (27). *miR-490-3p* overexpression in Breast cancer cells caused an inhibition of growth and invasiveness. This effect was determined by *miR-490-3p* targeting on tankyrase 2 (*TNKS2*) so blocking the activation of β-Catenin signaling (28). *miR-338-3p* targets Zinc finger E-box-binding homeobox 2 (*ZEB2*) causing an inhibition of NF-κB and PI3K/Akt signal pathways with a consequent reduction in cell growth and invasion (29). Described miRNAs have a tumor suppressor function in BC and are summarized in the Table 1. We also observed the upregulation of miRNAs in MpBC compared to metastatic patients, but we did not find studies in the literature demonstrating their involvement in breast carcinogenesis. Downregulated miRNAs in MpBC compared to the 3 metastatic patients can lead to greater expression of oncogenes in the tumor microenvironment, resulting in increased tumor growth in the MpBC patient. This could partly explain the worse prognosis of the MpBC case compared to metastatic patients in whom, less regulated by miRNAs, the genes involved in tumor growth are more expressed and able to perform freely their oncogenic function than in metastatic patients. Hence, it might be suggested that the study of the miRNA cargo can help to identify an expression profile that can be used to develop targeted delivery of exosome-based miRNAs. In EVs, pre-miRNAs can transform into mature miRNAs, thus enriching them and recovering a healthy microenvironment. By regulating gene expression, miRNA therapies may act on different targets usually not achieved from traditional treatments. In future studies, we will deepen the function and the mechanism of EV-contained miRNAs in carcinogenesis willing to improve MpBC therapies.

**TABLE 1:** Tumor suppressor miRNAs.

miRNA	Target	Function	References
miR-15a-5p	SALL4	Inhibition of cell proliferation, migration and invasion	(21)
miR-190	STC2	Inhibition of epithelial-mesenchymal transition and angiogenesis	(22)
miR-106b	MMP2	Inhibition of migration and invasion	(23)
miR-92b	EZH2	Inhibition of invasion	(24)
miR-1258	E2F	Inhibition of cell proliferation, migration, invasion	(25)
miR-26a	MCL-1	Inhibition of cell proliferation, migration, invasion	(26)
miR-30a	ROR1	Inhibition of epithelial-mesenchymal transition and metastasis	(27)
miR-490-3p	TNKS2	Inhibition of cell proliferation and invasion	(28)
miR-338-3p	ZEB2	Inhibition of cell proliferation and invasion	(29)

## Data Availability Statement

The datasets presented in this article are not readily available because of ethical and privacy reasons. Requests to access the datasets should be directed to IV, ivan.vannini@irst.emr.it.

## Ethics Statement

Ethical review and approval was not required for the study on human participants in accordance with the local legislation and institutional requirements. The patients/participants provided their written informed consent to participate in this study. Written informed consent was obtained from the individual(s) for the publication of any potentially identifiable images or data included in this article.

## Author Contributions

IV, TR, MPa, GG, GM, and FF contributed to the conceptualization and design of the study. IV, MU, and MM conduced the experiments. MU performed bioinformatic analysis. IA performed statistical analysis. MPu contributed to data collection. IV wrote the manuscript. All authors contributed to manuscript revision, read, and approved the submitted version.

## Conflict of Interest

The authors declare that the research was conducted in the absence of any commercial or financial relationships that could be construed as a potential conflict of interest.

## Publisher’s Note

All claims expressed in this article are solely those of the authors and do not necessarily represent those of their affiliated organizations, or those of the publisher, the editors and the reviewers. Any product that may be evaluated in this article, or claim that may be made by its manufacturer, is not guaranteed or endorsed by the publisher.

## Footnotes

1
https://cran.r-project.org/
